# The Finding of a Group IIE Phospholipase A_2_ Gene in a Specified Segment of *Protobothrops flavoviridis* Genome and Its Possible Evolutionary Relationship to Group IIA Phospholipase A_2_ Genes

**DOI:** 10.3390/toxins6123471

**Published:** 2014-12-18

**Authors:** Kazuaki Yamaguchi, Takahito Chijiwa, Naoki Ikeda, Hiroki Shibata, Yasuyuki Fukumaki, Naoko Oda-Ueda, Shosaku Hattori, Motonori Ohno

**Affiliations:** 1 Department of Applied Life Science, Faculty of Bioscience and Biotechnology, Sojo University, Kumamoto 860-0082, Japan; E-Mails: g1319d02@m.sojo-u.ac.jp (K.Y.); cavia_tschudii_xist@yahoo.co.jp (N.I.); mohno218@ybb.ne.jp (M.O.); 2 Medical Institute of Bioregulation, Research Center of Genetic Information, Kyushu University, Fukuoka 812-8582, Japan; E-Mails: hshibata@gen.kyushu-u.ac.jp (H.S.); yfukumak@gen.kyushu-u.ac.jp (Y.F.); 3 Department of Biochemistry, Faculty of Pharmaceutical Sciences, Sojo University, Kumamoto 860-0082, Japan; E-Mail: naoko@ph.sojo-u.ac.jp; 4 Institute of Medical Science, University of Tokyo, Oshima-gun, Kagoshima 894-1531, Japan; E-Mail: shattori@ims.u-tokyo.ac.jp

**Keywords:** group IIE phospholipase A_2_, venom, evolution, gene cluster, comparative genomics

## Abstract

The genes encoding group IIE phospholipase A_2_, abbreviated as IIE PLA_2_, and its 5' and 3' flanking regions of Crotalinae snakes such as *Protobothrops flavoviridis*, *P. tokarensis*, *P. elegans*, and *Ovophis okinavensis*, were found and sequenced. The genes consisted of four exons and three introns and coded for 22 or 24 amino acid residues of the signal peptides and 134 amino acid residues of the mature proteins. These IIE PLA_2_s show high similarity to those from mammals and Colubridae snakes. The high expression level of IIE PLA_2_s in Crotalinae venom glands suggests that they should work as venomous proteins. The blast analysis indicated that the gene encoding OTUD3, which is ovarian tumor domain-containing protein 3, is located in the 3' downstream of IIE PLA_2_ gene. Moreover, a group IIA PLA_2_ gene was found in the 5' upstream of IIE PLA_2_ gene linked to the OTUD3 gene (*OTUD3*) in the *P. flavoviridis* genome. It became evident that the specified arrangement of IIA PLA_2_ gene, IIE PLA_2_ gene, and *OTUD3* in this order is common in the genomes of humans to snakes. The present finding that the genes encoding various secretory PLA_2_s form a cluster in the genomes of humans to birds is closely related to the previous finding that six venom PLA_2_ isozyme genes are densely clustered in the so-called NIS-1 fragment of the *P. flavoviridis* genome. It is also suggested that venom IIA PLA_2_ genes may be evolutionarily derived from the IIE PLA_2_ gene.

## 1. Introduction

*Protobothrops* genus snakes (Crotalinae, Viperidae) are distributed in the southwestern islands of Japan, *P. flavoviridis* and *Ovophis okinavensis* in Amami-Oshima, Tokunoshima, and the Okinawa islands, *P. tokarensis* in the Tokara islands, and *P. elegans* in the Sakishima islands. The venoms of *Protobothrops* snakes are produced and stored in the venom glands, which are assumed to share an original developmental organ with the mammalian submaxillary glands. The injection of the venom through tubular front fangs causes various severe lesions in humans, such as myonecrosis, hemorrhage, and edema [1,2,3].

Phospholipase A_2_ (PLA_2_) [EC 3.1.1.4] catalyzes the hydrolysis of glycerophospholipid at the *sn-2* position to produce free fatty acids and lysophospholipids [4]. As various forms of PLA_2_s work in almost whole organs in the body [5], they are divided into three categories: secretory, cytosolic, and Ca^2+^-independent PLA_2_s, based on the working modes [6]. Furthermore, novel transcriptome analysis in mammals showed that secretory PLA_2_s are classified into 11 groups: IB, IIA, IIC, IID, IIE, IIF, III, V, X, XIIA, and XIIB, according to the primary structures and the organs to be expressed [7]. Snake venoms also contain PLA_2_ isoforms as major toxic components. With regard to the primary structures and the modes of disulfide bond pairings [8], snake venom PLA_2_s are classified into group IA found in Elapidae (Elapinae and Hydrophiinae) venoms and group II found in Viperidae (Viperinae and Crotalinae) venoms [9]. Group II venom PLA_2_s are further divided into group IIA PLA_2_s ([Asp^49^]PLA_2_ forms) and group IIB PLA_2_s ([Lys^49^]PLA_2_ forms) [10,11]. *P. flavoviridis* (Crotalinae) group IIA venom PLA_2_ genes form a multi-gene family of 16~32 copies per haploid [12] and are located at two loci on a microchromosome [13]. The mathematical analysis of their nucleotide sequences delineated that they have evolved in an accelerated manner to acquire isozymes with diverse physiological activities [14,15,16]. Recently, the nucleotide sequence of the 31,348 bp genome fragment of *P. flavoviridis* was completely deciphered. It showed that six PLA_2_ isozyme genes are aligned in series and four of them are linked with the fragment of CR1 long interspersed nuclear element (LINE), named PcRTF (PLA_2_ gene-coupled reverse transcriptase fragment), at the 3' terminus [13]. We call this fragment, composed of six PLA_2_ isozyme genes, NIS-1 (Figure 5 and Figure 6). Fry *et al.* (2012) found that group IIE PLA_2_ was expressed in the venom glands of Colubridae snakes and proposed that it is a component of Colubridae snake venoms [17].

In the work reported here, we sequenced the segment-harboring novel IIE PLA_2_ gene linked to *OTUD3*, which codes for the ovarian tumor domain-containing protein (OTUD) 3, in the *P. flavoviridis* genome. Moreover, the IIA PLA_2_ gene was found in the 5' upstream of IIE PLA_2_ gene. It became evident that the linear arrangement of the IIA PLA_2_ gene, the IIE PLA_2_ gene, and *OTUD3*, in this order, is common in the genomes of humans to snakes. It is also found that the clusters of the genes encoding various PLA_2_s in the 5' upstream region of IIE PLA_2_ gene in the genomes of humans to birds possibly correspond to those of six PLA_2_ isozyme genes in NIS-1 fragment of *P. flavoviridis* genome. Possible conversion of the IIE PLA_2_ gene to the IIA PLA_2_ gene and its multiplication in Crotalinae snake genomes are discussed.

## 2. Materials and Methods

### 2.1. Materials

*P. flavoviridis* (Amami-Oshima Island, Japan), *P. tokarensis*, *P. elegans*, and *O. okinavensis* specimens were provided from the Institute of Medical Sciences of the University of Tokyo. High molecular weight genomic DNAs were prepared from the livers or the venom glands of the snakes according to the method of Blin and Stafford (1976) [18]. Total RNAs were prepared from various organs of the snakes according to the protocol of ISOGEN (Nippon Gene, Toyama, Japan). Restriction endonucleases and KOD plus DNA polymerase were purchased from Nippon Gene and TOYOBO (Osaka, Japan), respectively. The other reagents and antibiotics were from Nacalai Tesque (Kyoto, Japan) and TAKARA BIO (Shiga, Japan). Specific oligonucleotide primers were synthesized by GENNET (Fukuoka, Japan). All relevant ethical safeguards have been met in relation to animal experimentation.

### 2.2. Cloning and Sequencing of the Genome Segments Harboring IIE PLA_2_ Gene and Its 5' and 3' Flanking Regions of Crotalinae Snakes and of Their IIE PLA_2_ cDNAs 

**Figure 1:**
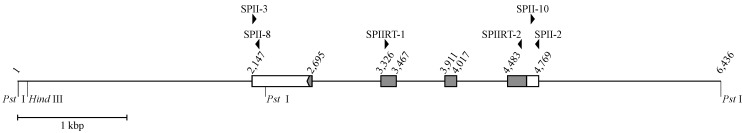
The schematic representation of the genome segment harboring the Crotalinae IIE PLA_2_ gene. The nucleotide positions are numbered. Closed boxes represent open reading frames (ORFs) and open boxes untranslated regions (UTRs). Vertical bars indicate the positions of restriction enzyme sites. Arrow heads show the positions of primers.

The personal expressed sequence tags (ESTs) database was constructed to unite the snake ESTs collected from Genbank and the ESTs of *P. flavoviridis* venom glands supplied from the Medical Institute of Biodefense of Kyushu University (Fukumaki and Shibata, unpublished) by utilizing the NCBI C++ Toolkit (National Center for Biotechnology Information, Bethesda, MD, USA). The tblastx analysis of the database was carried out with the nucleotide sequences of *Homo sapiens* IIE PLA_2_ gene (NM_014589) [19] and *Mus musculus* IIE PLA_2_ gene (NM_012044) [20] as query. The 1085 bp candidate subject, named isotig03504, was acquired. Isotig is a subsequence of an isogroup. An isogroup is an assembled transcription sequence approximately equivalent to that of a gene. Based on the nucleotide sequences of its 5' and 3' ends, the sense primer named SPII-3, 5'-gTA gAC TgC gCg TAA TTT gTA g-3', and the antisense primer named SPII-2, 5'-ggC CgA gTC CgT CgT AgC T-3', were designed (Figure 1). Genomic PCRs with these primers were carried out against the *P. flavoviridis*, *P. tokarensis*, *P. elegans*, and *O. okinavensis* genomes as the templates. Thus, about 2.6 kbp DNA fragments, which contain four exons coding for IIE PLA_2_s, were obtained. Moreover, to acquire the nucleotide sequences of its 5' and 3' flanking regions, adaptor ligation PCR, designated as “Ligation-Mediated PCR (LM-PCR)” (Takara Bio, Shiga, Japan), was conducted. The genomic DNA obtained by digestion with *Hin*d III or *Pst* I was ligated with the adaptor nucleotide fragments with *Hin*d III- or *Pst* I-terminus, designated as a “cassette.” Then, PCR was done with a C1 primer, 5'-gTA CAT ATT gTC gTT AgA ACg CgT AAT ACg ACT CA-3', which can anneal to the “cassettes,” and SPII-2 primer to amplify the genome fragment containing the 5' flanking region or SPII-3 primer to amplify the genome fragment containing the 3' flanking region (Figure 1). Moreover, to ensure the validity of the PCR, another PCR was conducted with the C2 primer, 5'-CgT TAg AAC gCg TAA TAC gAC TCA CTA TAg ggA gA-3', which can anneal to the “cassette” at the internal portion of the C1 primer and SPII-8 primer, 5'-CAg TCC TTC CAT AAA gCT C-3', to amplify the genome fragment corresponding to the 5' flanking region, or SPII-10 primer, 5'-CTT gCA CgT CTC Cgg ATT gTg-3', to amplify the genome fragment corresponding to the 3' flanking region to be overlapped to the fragments prepared as described above (Figure 1). Amplified genome fragments were ligated to pCR™-Blunt II-TOPO^®^ vector (Life Technologies, Carlsbad, CA, USA), and transformed with DH5α competent cells (Takara Bio). The nucleotide sequences were determined with an ABI 3130xl capillary sequencer. The nucleotide sequences of Crotalinae IIE PLA_2_ genes and their 5' and 3' flanking regions are available in the Genbank/EMBL/DDBJ databases under Accession Nos. KM488538-KM488542.

### 2.3. Acquisition of the Genome Segment Harboring IIA PLA_2_ Gene, IIE PLA_2_ Gene, and OTUD3 of P. flavoviridis

Long genomic PCR was carried out with CHO5, 5'-gAT TCg ggA ggA TgA ggA CTC TC-3' [21], which anneals to the 5' UTR of the IIA PLA_2_ gene, and OTUD3-1, 5'-CCT Tgg TAg CCT CTT TgC CAT CAg-3', which anneals to the middle portion of intron 7 of *OTUD3*, against the *P. flavoviridis* genome in order to confirm whether the IIA PLA_2_ gene is located in the 5' upstream of the IIE PLA_2_ gene linked to *OTUD3*.

### 2.4. Expression Analysis by Semi-Quantitative RT-PCR of Crotalinae IIE PLA_2_ mRNA

The first strand cDNA of snake body organs was synthesized by reverse transcription and primer extension of the SMART cDNA Library Construction Kit (Clontech Laboratories, Mountain View, CA, USA). Based on the nucleotide sequences of the genes encoding the IIE PLA_2_s of Crotalinae snakes, the sense primer SPIIRT-1, 5'-CAC ATC ATC RAg CAC TTg AC-3', which commonly anneals to the middle portion of exon 2, was designed. The antisense primers SPIIRT-2 (5'-TCC TTC gCA CAg gCg gTT A-3', which can anneal specifically to the middle portion of exon 4 of the *P. flavoviridis* IIE PLA_2_ gene) and SPIIRT-3 (5'-TCC TTC gCA CAg gCg gTT A-3', which can anneal specifically to the middle portion of exon 4 of the *O. okinavensis* IIE PLA_2_ gene) were designed. The cDNA of β-actin, designated as ACTB, was amplified as an internal standard with the sense primer SHU7, 5'-CAg AgC AAg AgA ggT ATC C*N*-3' (*N* = G, A, T, C), and the antisense primer SHU8, 5'-TAg ATg ggC ACA gTg Tgg gN-3', as described previously [22]. The intensities of the bands of the amplified DNA fragments were estimated with Image J (NIH, Bethesda, MD, USA) and corrected relative to those of ACTB. The vertical numerals of the histogram are the values relative to that of the lung of *P. flavoviridis*, taken as one.

### 2.5. Phylogenetic Analysis of Secretory PLA_2_s

A phylogenetic tree was constructed based on the amino acid sequences of the mature proteins of the secretory PLA_2_s from various organisms (*Homo sapiens*, *Mus musculus*, *Gallus gallus*, *Ornithorhynchus anatinus*, *Macaca mulatta*, *Pan troglodytes*, *Oryctolagus cuniculus*, *Canis lupus familiaris*, *Bos taurus*, *Laticauda semifasciata*, *Leioheterodon madagascariensis*, *Dispholidus typus*, *P. flavoviridis*, *P. tokarensis*, *P. elegans*, and *O. okinavensis*) with the maximum likelihood method of the RAxML program [23]. The degrees of confidence for internal lineage in the phylogenetic tree were determined by the bootstrap confidence [24] using Kimura’s (1969) method to compute a distance matrix with 1000 replicates [25].

### 2.6. Comparative Structural Analysis of the Cluster Domains of Secretory PLA_2_ Genes in the Genomes

The BLAST analysis done with the nucleotide sequences of the cDNA encoding human secretory IIA, IIC, IID, IIE, IIF, and V PLA_2_s [5], against the draft genome databases of *H. sapiens* (GRCh37P.p13), *M. musculus* (GRCm38.p2), and *G. gallus* (Gallus_gallus-4.0), deciphered that secretory PLA_2_s are distributed within a 300 kb genome segment of *H. sapiens* chromosome 1 (NC_000001 GPC_000000025), 200 kb of *M. musculus* chromosome 4 (NC_000070 GPC_000000777), and 21 kb of *G. gallus* chromosome 21 (NC_006108 GPC_000000738) (Figure 5). The *OTUD3* (NP_056022 for *H. sapiens*, NP_082729 for *M. muculus*, XP_424363 for *G. gallus*) was found in the 3' downstream of a series of PLA_2_ genes in these regions. In the case of *Ophiophagus hannah*, all the draft genome data (AZIM00000000.1) [26] were downloaded and made it personal *O. hannah* genome database. Referring to these gene arrangements in *H. sapiens*, *M. musculus*, and *G. gallus*, the contig harboring secretory PLA_2_ genes was constructed through tblastn analysis against the *O. hannah* personal genome database. Then, the chromosomal loci of the secretory PLA_2_ genes and *OTUD3* were mapped on their scaffolds and compared with the *P. flavoviridis* genome segments harboring the IIE PLA_2_ gene and *OTUD3* (this study) and the NIS-1 fragment composing of six consecutive IIA PLA_2_ genes [13].

## 3. Results and Discussion

### 3.1. The Structure of a 6436 bp P. flavoviridis Genome Segment Containing the IIE PLA_2_ Gene

The tblastx analysis of the personal EST database gave three subjects, two of which were the wrong transcripts; one contained a stop codon with the redundant mutations and the other was fused with an irrelevant nucleotide fragment. As the deduced amino acid sequence encoded by the remaining subject, isotig03504, is similar to those of human (59%) and mouse IIE PLA_2_ proteins (61%)—in particular, the positions of the half-cystine residues and the sequences of the Ca^2+^ binding site and the catalytic site are identical—this subject is thought to be the transcript derived from the gene encoding *P. flavoviridis* IIE PLA_2_, designated as *Pf*IIEPLA_2_. Genomic PCR with SPII-2 and SPII-3 primers, which can anneal to the 5' and 3' terminal portions, respectively, of isotig03504, of the Amami-Oshima *P. flavoviridis* genome gave a 2616 bp fragment, which covers from the 5' portion of the first exon to the 3' terminal portion of the fourth exon of the *Pf*IIEPLA_2_ gene. In addition, LM-PCR of the Amami-Oshima *P. flavoviridis* genome gave a 6436 bp genome fragment harboring the *Pf*IIEPLA_2_ gene and its 5' and 3' flanking regions (Figure 1). Moreover, genomic PCR of *P. tokarensis*, *P. elegans*, and *O. okinavenesis* genome DNA also gave the genome fragments harboring the *Pt*IIEPLA_2_, *Pe*IIEPLA_2_, and *Oo*IIEPLA_2_ genes, together with their 5' and 3' flanking regions.

### 3.2. The Characteristic Primary Structures of Snake IIE PLA_2_ Proteins

**Figure 2:**
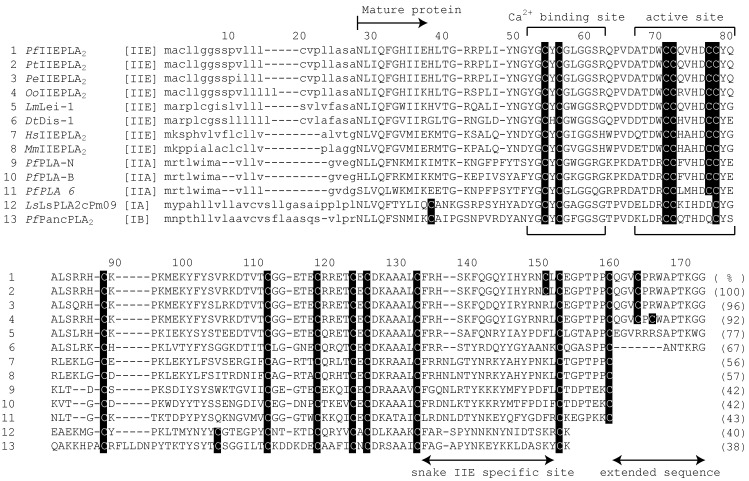
The aligned amino acid sequences of IIE, IIA, IA and IB PLA_2_s from snakes and mammals. The positions are numbered from the first residue of the signal peptides. The half-cystines are shown in shaded letters. Abbreviations: *Dt*, *Dispholidus typus*; *Hs*, *Homo sapiens*; *Lm*, *Leioheterodon madagascariensis*; *Ls*, *Laticauda semifasciata*; *Mm*, *Mus musculus*; *Oo*, *Ovophis okinavensis*; *Pe*, *Protobothrops elegans*; *Pf*, *P. flavoviridis*; and *Pt*, *P. tokarensis*. References: *Pf*IIEPLA_2_ (this work); *Pt*IIEPLA_2_ (this work); *Pe*IIEPLA_2_ (this work); *Oo*IIEPLA_2_ (this work); *Dt*Dis-1 (AFH66958) [17]; *Hs*IIEPLA_2_ (NP_055404) [19]; *Lm*Lei-1 (AFH66960) [17]; *Ls*LsPLA2cPm09 (BAB03302) [32]; *Mm*IIEPLA_2_ (NP_036174) [20]; *Pf*PLA-B (BAG82670) [13]; *Pf*PLA-N (BAG82669) [13]; *PfPLA 6* (BAJ84552) [29]; and *Pf*PancPLA_2_ (BAN08536) [31]. Numerals in parentheses show the identities of the amino acid sequences against those of the mature protein of *Pf*IIEPLA_2_.

**Figure 3:**
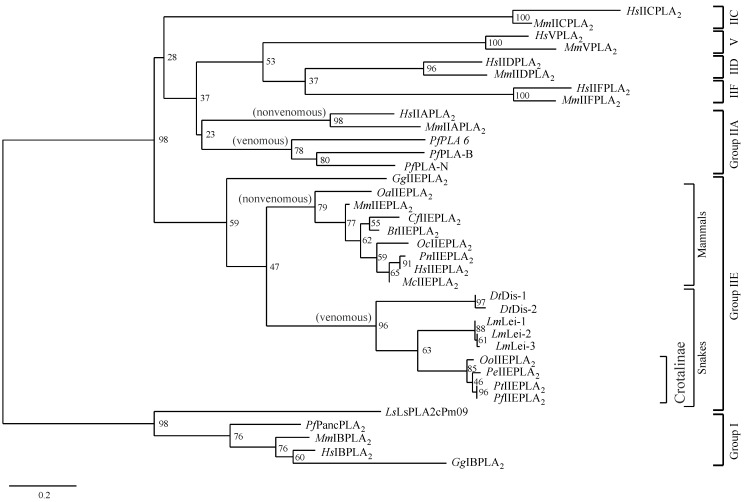
The phylogenetic tree constructed for the secretory PLA_2_s of snakes and mammals, based on the amino acid sequences of their mature proteins. The numerals at the nodes represent bootstrap confidence values and the branch lengths represent the numbers of amino acid substitutions per site. Abbreviations: *Bt*, *Bos taurus*; *Cf*, *Canis lupus familiaris*; *Gg*, *Gallus gallus*; *Mc*, *Macaca mulatta*; *Oa*, *Ornithorhynchus anatinus*; *Oc*, *Oryctolagus cuniculus*; and *Pn, Pan troglodytes*. References: *Bt*IIEPLA_2_ (NP_001179015) [33], *Cf*IIEPLA_2_ (XP_544525) (automated computational prediction by GNOMON); *Dt*Dis-2 (AFH66959) [17]; *Gg*IBPLA_2_ (NP_001138961) [34]; *Gg*IIEPLA_2_ (NP_001171878) [35]; *Hs*IBPLA_2_ (NP_000919) [36]; *Hs*IIAPLA_2_ (NP_001155199) [37]; *Hs*IICPLA_2_ (NP_001099042) [38]; *Hs*IIDPLA_2_ (NP_036532) [39]; *Hs*IIFPLA_2_ (NP_073730) [40]; *Hs*VPLA_2_ (NP_000920) [38]; *Lm*Lei-2 (AFH66961) [17]; *Lm*Lei-3 (AFH66962) [17]; *Mc*IIEPLA_2_ (XP_001094364) (automated computational prediction by GNOMON); *Mm*IBPLA_2_ (NP_035237) [41]; *Mm*IIAPLA_2_ (NP_001076000) [42]; *Mm*IICPLA_2_ (NP_032894) [41]; *Mm*IIDPLA_2_ (NP_035239) [39]; *Mm*IIFPLA_2_ (NP_036175) [20]; *Mm*VPLA_2_ (NP_001116426) [41]; *Oa*IIEPLA_2_ (XP_001505559) (automated computational prediction by GNOMON); *Oc*IIEPLA_2_ (XP_002716050) (automated computational prediction by GNOMON); *Oo*IIEPLA_2_ (this work); *Pe*IIEPLA_2_ (this work); *Pf*IIEPLA_2_ (this work); *Pn*IIEPLA_2_ (XP_001163677) (automated computational prediction by GNOMON); and *Pt*IIEPLA_2_ (this work).

The deduced amino acid sequences of Crotalinae IIE PLA_2_s are aligned with those of the IIE PLA_2_s from two Colubridae genus snakes [17], from *H. sapiens* (NP_055404) [19] and *M. musculus* (NP_036174) [20], as well as those from the venom IIA PLA_2_s from *P. flavoviridis* [27,28,29], venom IA PLA_2_ from *Laticauda semifasciata* [30], and pancreatic IB PLA_2_ from *P. flavoviridis* [31] (Figure 2). This alignment confirms that four PLA_2_s, *Pf*IIEPLA_2_, *Pt*IIEPLA_2_, *Pe*IIEPLA_2_, and *Oo*IIEPLA_2_, from Crotalinae genus snakes are clearly classified into group IIE. Although the amino acid sequences of IIE and IIA PLA_2_s are similar to one another, the *C*-terminal amino acid sequences from the 133th residue are distinct between them. The phylogenetic analysis including other group PLA_2_s, such as IA, IB, IIC, IID, IIF, and V PLA_2_s, also shows that the IIE PLA_2_s, including four novel Crotalinae PLA_2_s, form an independent clade separated from other group PLA_2_s (Figure 3). Moreover, IIE PLA_2_s are further divided into those from snakes or mammals, in accordance with the differences in their *C*-terminal sequences.

### 3.3. Venom Gland-Specific Expression of IIE PLA_2_s in Crotalinae Snakes

**Figure 4:**
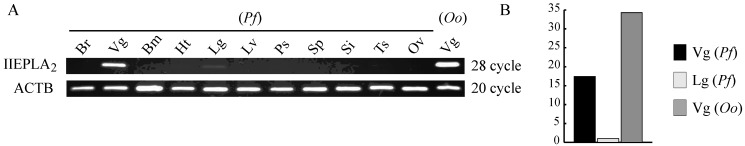
(**A**) Electrophoretograms of RT-PCR products of IIE PLA_2_ mRNA and β-actin mRNA (ACTB, as the internal standard) for various organs of *P. flavoviridis* and *O. okinavensis*; (**B**) The histogram showing the relative intensities of the bands of IIE PLA_2_s from (**A**). Abbreviations: Bm, Buccinator muscle; Br, Brain; Ht, Heart; Lg, Lung; Lv, Liver; Ov, Ovary; Ps, Pancreas; Si, Small intestine; Sp, Spleen; Ts, Testis; and Vg, Venom gland.

In general, mammalian IIE PLA_2_s are non-venomous somatic molecules. On the other hand, as mRNA-encoding IIE PLA_2_s, abbreviated as Lei-1, 2, and 3 and Dis-1 and 2, were found in the venom glands of Colubridae snakes *Leioheterodon madagascariensis* and *Dispholidus typus*, respectively, it was proposed that the IIE PLA_2_ of Colubridae snakes may work as a venom protein [17]. In the case of Crotalinae genus snakes, the expression analysis by semi-quantitative RT-PCR performed on several organs of *P. flavoviridis* and *O. okinavensis* showed that the IIE PLA_2_s of *P. flavoviridis* and *O. okinavensis* are expressed at remarkably high levels in the venom glands (Figure 4). These results suggest that Crotalinae IIE PLA_2_s are also venom proteins. Its expression in the lungs, though at a low level, may show that they act as an immune factor to neutralize bacteria infected through the air [43,44].

### 3.4. The Genome Structures Harboring Secretory PLA_2_ Genes Are Conserved from Human to Snake

**Figure 5:**
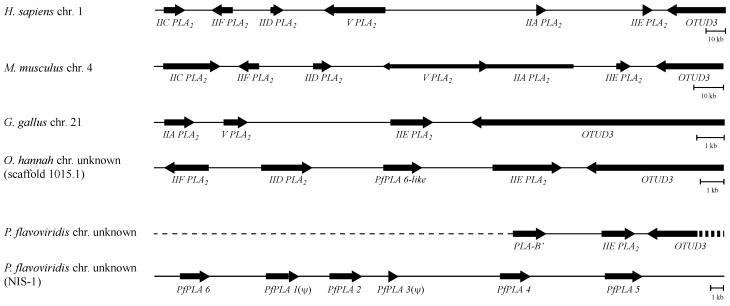
Diagrammatic representation of secretory PLA_2_ genes in human, mouse, chicken, and snake genomes. The names of the organisms and the numbers of chromosomes are shown at left. Bold arrows indicate the areas of the genes in the chromosomes and the direction of arrows indicates the transcribing direction of the genes. Dashed lines indicate the regions where the nucleotide sequences are not determined. Organisms and genome information: *H. sapiens* chr. 1 (NC000001.10); *M. musculus* chr. 4 (NC000070.6); *G. gallus* chr. 21 (NC006108.3); *O. hannah* scaffold 1015.1 (AZIM01001014); *P. flavoviridis* NIS-1 (AB440236), *PfPLA 6* (AB588615), and *Pf*IIEPLA_2_ (this work, KM488539).

The BLAST analysis showed that *OTUD3* is located in the 3' downstream of the *Pf*IIEPLA_2_ gene in the *P. flavoviridis* genome (Figure 5). Based on the linear arrangement of the IIE PLA_2_ gene and *OTUD3* and the nucleotide sequences of the cDNA encoding human secretory IIA, IIC, IID, IIE, IIF, and V PLA_2_s [5], BLAST analysis was made against the *H. sapiens* draft-genome database [45]. Then, it was found that the secretory PLA_2_ genes are aligned in the 5' upstream of *OTUD3* within the 300-kb genome segment of *H. sapiens* chromosome 1. Interestingly, similar genome structures were found in the *M. musculus*, *G. gallus*, and *O. hannah* genomes (Figure 5). Particularly, it should be noted that the linear arrangement of the IIA PLA_2_ gene, the IIE PLA_2_ gene, and *OTUD3*, that is, the triplet genes, in this order is common in the genomes of human, mouse, and snake. In the case of *O. hannah*, it was found that three PLA_2_ genes, that is, the *Pf*PLA 6-like gene, the IID PLA_2_ gene, and the IIF PLA_2_ gene, are aligned in the 5' upstream of the IIE PLA_2_ gene and *OTUD3*. The *Pf*PLA 6 gene is contained in the *P. flavoviridis* NIS-1 fragment [13,29]. In the case of the *G. gallus* genome, the IIA PLA_2_ gene is found in the 5' upstream of IIE PLA_2_ gene, but the IIA and IIE PLA_2_ genes are interrupted by the V PLA_2_ gene. This unexpected location of the V PLA_2_ gene in *G. gallus* is thought to be specific to birds. The alignment of secretory PLA_2_ genes in the 5' upstream of *OTUD3* should be highly conserved among the vertebrates. In this work, we also acquired an 11 kb genome fragment of *P. flavoviridis*, which encompasses from the gene encoding venom IIA PLA_2_, called PLA-B', at the 5' terminus to intron 7 of *OTUD3* at the 3' terminus, by genomic PCR with CHO5, which anneals to the 5' UTR of the venom IIA PLA_2_ gene, and OTUD3-1, which specifically anneals to the middle portion of intron 7 of *OTUD3* (data not shown). The alignment of six IIA PLA_2_ isozyme genes in the *P. flavoviridis* NIS-1 fragment is shown in Figure 5 [13]. *PfPLA 6* codes for a novel basic [Asp^49^]PLA_2_ [29], *PfPLA 1*(Ψ) is 91% similar in sequence to *PfPLA 6* with 10 nucleotide deletions, *PfPLA 2* [Lys^49^]PLA_2_ called BPII, *PfPLA 3*(Ψ) is a fragment from the second intron to the fourth exon of the G6D49PLA_2_ gene found in the *Trimeresurus stejnegeri* snake [46], *PfPLA 4* is a neurotoxic [Asp^49^]PLA_2_ called PLA-N, and *PfPLA 5* is a basic [Asp^49^]PLA_2_ called PLA-B. PLA-B and PLA-B' are the same isozymes with only one amino acid substitution at position 53, Glu or Gly, respectively [47,48]. It could be assumed that a cluster of IIA PLA_2_ isozyme genes like NIS-1 is located in the 5' upstream of the triplet genes, that is, the PLA-B' gene, the IIE PLA_2_ gene, and *OTUD3*, in the *P. flavoviridis* genome.

### 3.5. The Structural Relationship between the IIE PLA_2_ Gene and IIA PLA_2_ Genes in the P. flavoviridis Genome

**Figure 6:**
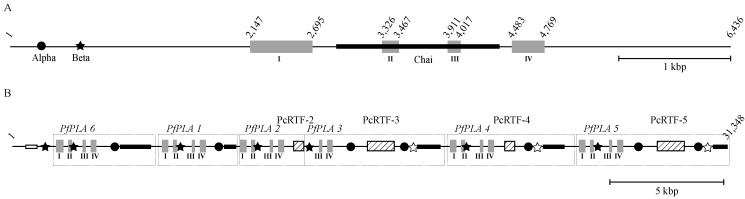
The schematic representation of the locations of three typical nucleotide segments, named Alpha, Beta, and Chai, in the *Pf*IIEPLA_2_ gene (**A**); and in six IIA PLA_2_ genes in the NIS-1 fragment [13,29] of *P. flavoviridis* (**B**). Alpha, Beta, and Chai segments are shown by closed circle, closed star, and closed box, respectively. Gray boxes indicate exons of the PLA_2_ gene and their numbers are shown as Roman numerals below the boxes. Boxes filled with oblique lines indicate the retroelements named PcRTFs [13]. The nucleotide position numbers are the same as those in Figure 1 and those reported previously [13]. The open star and open box mean the antisense nucleotide segments of Beta and Chai segments, respectively. The genome fragment, which encompasses from the venom IIA PLA_2_ isozyme genes with or without PcRTF segment in the 3' terminus to the Alpha and Chai segments, is bracketed as a unit.

Two-BLAST analysis showed that the three highly homologous nucleotide segments, named Alpha, Beta, and Chai, are commonly contained in both the *Pf*IIEPLA_2_ gene (Figure 6A) and venom IIA PLA_2_ isozyme genes clustered in the NIS-1 fragment (Figure 6B). Alpha, Beta, and Chai segments are about 0.4, 0.3, and 1.4 kbps in length with 69%–94% aligned scores. Their locations in the genes are distinctive. In the *Pf*IIEPLA_2_ gene (Figure 6A), the Alpha segment is found in the 5' flanking region, the Beta segment in the 3' downstream of the Alpha segment in the 5' flanking region, and the Chai segment encompasses from the middle portion of intron 1 to the posterior portion of intron 3. The NIS-1 fragment consists of a series of IIA PLA_2_ isozyme genes with or without PcRTF segment in the 3' terminus, each of which is bracketed as a unit in Figure 6B. Here, the Alpha and Chai segments are located in the 3' flanking region in this order and the Beta segment is in the anterior portion of intron 2 (Figure 6B). Therefore, it could be thought that after the prototype of venom IIA PLA_2_ gene containing the three segments had been formed, its multiplication occurred as seen in NIS-1 fragment. Since the Alpha, Beta, and Chai segments are found at the particular locations, it is hard to imagine that the three segments had been introduced after multiplication of the venom IIA PLA_2_ genes. On the other hand, it could be thought that the IIA PLA_2_ gene had been converted from a IIE PLA_2_ gene as a precursor with unknown mechanism.

**Figure 7:**
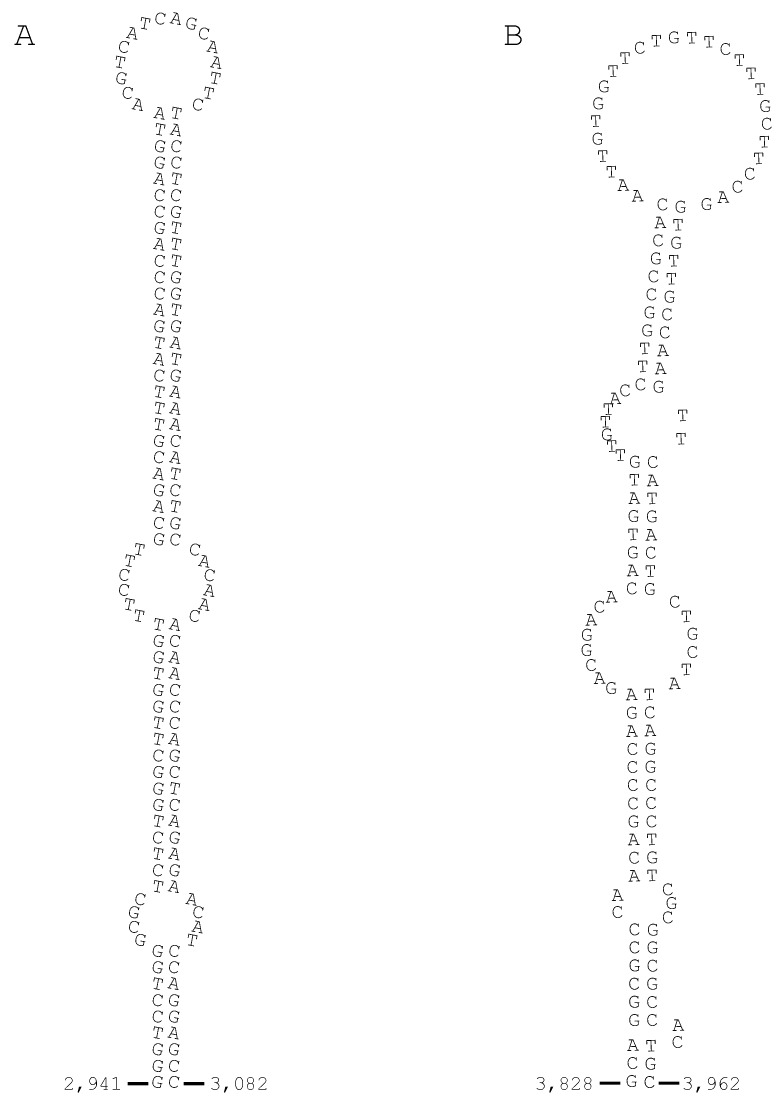
The constructed stem-loop structures of Chai-1 (**A**) and Chai-2 segments (**B**). The secondary structures are deduced based on their nucleotide sequences via DNA folding form of the mfold Web Server. The numerals at both termini of the segments are the position numbers of the corresponding nucleotides in Figure 6A.

### 3.6. Different Multiplication Processes between Non-Venomous Secretory PLA_2_ Genes and P. flavoviridis Venom IIA PLA_2_ Genes

As *OTUD3* is a single-copy gene and codes for an ordinary non-venomous protein, structural and functional boundaries must exist between the IIE PLA_2_ gene and *OTUD3*. The genome domain harboring the cluster of various PLA_2_ genes seems to be easily multiplied, unlike that harboring *OTUD3*. Thus, it could be assumed in the human to snake genomes that a series of secretory PLA_2_ genes have multiplied toward the 5' upstream direction from the IIE PLA_2_ gene as the ancestor and diversified to various PLA_2_ gene species (Figure 5). However, the multiplication pattern of *P. flavoviridis* venom IIA PLA_2_ isozyme genes in the NIS-1 fragment is considerably different from those of non-venomous secretory PLA_2_ genes. The IIA PLA_2_ isozyme genes of the NIS-1 fragment are periodically and densely repeated, whereas the non-venomous secretory PLA_2_ genes are considerably scattered and the proteins encoded are structurally diversified so as to be classified into IIA, IIC, IID, IIF, and V PLA_2_s (Figure 5). The two mechanisms may be considered for multiplication of PLA_2_ genes. As the two nucleotide sequences in Chai segments, named Chai-1 and Chai-2, can be predicted to form stem-loop structures (Figure 7A,B), which could be the scaffolding of the gene recombination [49,50], it appears that such gene recombination might have been involved in the multiplication of non-venomous secretory PLA_2_ genes. On the other hand, Castoe *et al.* (2011) pointed out that the quantities of retroelements like SINEs and LINEs in venomous snake genomes are much higher than those in nonvenomous snake genomes [51]. In fact, the associated forms between PLA_2_ genes and CR1 LINEs were found in the *P. flavoviridis* NIS-1 fragment as mentioned above [13]. This suggests that retrotransposition, such as 3'-transduction [52,53], with CR1 LINE has participated in the multiplication of venom IIA PLA_2_ isozyme genes.

## Abbreviations

CR 1: chicken repeat 1
EST: expressed sequence tag
LINE: long interspersed nuclear element
OTUD3: ovarian tumor domain-containing protein 3
*Oo*: *Ovophis okinavensis*
ORF: open reading frame
*P*: *Protobothrops*
PLA_2_: phospholipase A_2_
SINE: short interspersed nuclear element
UTR: untranslated region
